# A Cross-Sectional Survey of Swedish Primary Healthcare Nurses' Discontent With Their Current Job

**DOI:** 10.1155/2024/2786600

**Published:** 2024-09-30

**Authors:** Elin Karlsson, Nadine Karlsson, Hanna Fernemark, Ida Seing, Janna Skagerström, Emma Brulin, Per Nilsen

**Affiliations:** ^1^ Department of Health, Medicine and Caring Sciences Division of Health and Society Linköping University, Linkoping SE-581 83, Sweden; ^2^ Primary Healthcare Centre, Lambohov, Linkoping, Sweden; ^3^ Department of Behavioral Science and Learning Linkoping University, Linkoping, Sweden; ^4^ Research and Development Unit in Region Ostergötland, Linkoping, Sweden; ^5^ Unit of Occupational Medicine Institute of Environmental Medicine Karolinska Institutet, Stockholm, Sweden; ^6^ School of Health and Welfare Halmstad University, Halmstad, Sweden

**Keywords:** change fatigue, job satisfaction, leadership, social support, turnover intentions, work environment, working conditions

## Abstract

Nursing staff turnover is an increasing problem for healthcare globally. In Sweden, the shortage of nurses in primary healthcare has increased significantly in recent years. This development is alarming because primary healthcare, both in Sweden and internationally, is responsible for a large part of healthcare. The aim of this study was to explore working conditions (change fatigue, leadership climate, and social support from colleagues) and characteristics of primary care nurses who are discontent with their current job, i.e., those with high turnover intentions and poor job satisfaction in Sweden. This was a cross-sectional survey of 466 registered nurses working in Swedish primary healthcare. Data were analyzed using descriptive statistics and logistic regression. The results demonstrate that 21.1% of the responding nurses are discontent with their current job and have considered quitting. Being discontent had significant associations with poor leadership climate (*p* < 0.001), lack of social support from colleagues (*p* < 0.001), change fatigue (*p* < 0.001), poor health (*p* < 0.001), and working more than 40 h per week (*p*=0.02). The results have implications for how healthcare organizations structure the work of nurses in primary healthcare and how they can attract and retain future staff to these workplaces.

## 1. Introduction

Nursing staff turnover is an increasing problem for healthcare globally. The World Health Organization estimates that there is a global shortage of 5.9 million nurses [1]. In Sweden, the National Board of Health and Welfare has identified a severe national nursing shortage [2, 3], and the demand for nurses in the healthcare system has increased sharply in recent years [4]. The proportion of primary healthcare nurses per 1000 population in Sweden has decreased since the early 2000s [5]. There are reports that the availability of nurses in primary healthcare does not reach actual needs [6]. This development is particularly alarming as primary healthcare is responsible for a large part of healthcare. Furthermore, primary healthcare in Sweden is expected to take on an important role in the development of an integrated care model to achieve a more holistic form of care to prevent disease, manage chronic conditions, bring care closer to home, and educate the population on better self-care [7].

Nurses leave healthcare for numerous reasons, including dissatisfaction with psychosocial working conditions such as staffing, heavy workload, low wages, poor leadership, lack of recognition, and inadequate authority [8, 9]. Turnover intention is an employee's willingness to voluntarily quit their job [10], and it has been found to correlate with the actual turnover [11]. Studies have shown that the turnover intention is associated with sickness absenteeism, reduced job performance, low work commitment [12], and low job satisfaction [13, 14]. Job satisfaction—i.e., the degree to which an employee enjoys their job or various aspects of their job [15]—correlates with nurses' self-confidence, communication, and mental health [16], as well as with patient care quality [17]. In addition, high job satisfaction is a major contributory factor to the intention to stay in the nursing profession [18, 19].

While nurses can have the intention to leave their jobs, they may still be satisfied with working as a nurse. Thus, they may express a desire to quit their job to look for other employment that can facilitate their personal development and provide better career opportunities. There may also be practical reasons, such as commuting distance or family circumstances. Still, there is a well-established negative relationship between turnover intention and job satisfaction; the more job satisfaction decreases, the more turnover intention increases [20, 21]. Turnover intention is also correlated with numerous factors besides job satisfaction, e.g., sickness absenteeism and reduced job performance [12]. However, the combined measure ascertains that low job satisfaction is a contributing factor to high turnover intention, which is important because job satisfaction plays a crucial role in attracting and retaining quality nurses and preventing the departure of discontent nurses.

Nurses' turnover and job satisfaction may also be adversely affected by many organizational changes that primary care has undergone in recent decades that have made nursing an increasingly complex profession [9]. Aging populations, changing disease patterns, new treatments for diseases, technological advancements (including telemedicine), an increased emphasis on patient/provider relationships based on partnership and mutual empowerment, political reforms, and policy initiatives all place strong demands on primary care organizations and health professionals' capacity to implement change [22–26]. Research has shown that organizational changes are often associated with employees' psychological uncertainty about how the changes will affect them [27–29]. Studies on nurses [30, 31] have shown that workplace changes can lead to change fatigue, which is the overwhelming feeling of stress, exhaustion, and burnout associated with rapid and continuous change in the workplace [30]. It is therefore useful to explore whether change fatigue is more common among nurses who are discontent.

Social support received at the workplace, as well as the level of leadership, are important factors in the work environment that may act as buffers for some of the negative aspects previously described. For example, social support from colleagues in a workplace is an important predictor of increased intention to stay in an organization and in improved job satisfaction [32]. Other studies on the social support of nurses in primary healthcare demonstrate that an unsupportive clinical environment is associated with lower ratings in quality of care [33]. Social support can be broadly defined as the assistance and protection given by others [34]. Studies have shown that there is a positive relationship between nurses' social support in the workplace and the quality of patient care [35–38]. Similarly, leadership is positively associated with an intention to stay and job satisfaction, respectively [39, 40]. Leadership is defined as the art of influencing others to achieve their maximum potential to accomplish any task, objective, or project [41, 42]. Leadership climate refers to the way employees perceive leadership in terms of the manager's ability to create mutual trust and the emotional tone that characterizes the workplace [43].

Despite their obvious relevance for nursing retention and recruitment, there is limited research on primary care nurses' psychosocial working conditions in terms of turnover intention, job satisfaction, change fatigue, social support, and leadership climate. These issues have been studied primarily in hospital care settings [44]. Turnover intention and job satisfaction have been examined in primary healthcare nurses in Ireland [45], the United States [46], and South Africa [47]; job satisfaction has been studied among Canadian primary healthcare nurses [48]; and turnover intention and professional support have been investigated among primary healthcare nurses in Scotland [49]. No previous study has explored the working conditions and characteristics of primary care nurses who are discontent with their current job. This study combines high turnover with poor job satisfaction in order to examine nurses' discontent with their current job.

This study was developed to address numerous knowledge gaps. The aim was to explore working conditions (change fatigue, leadership climate, and social support from colleagues) and characteristics of primary care nurses who are discontent with their current job, i.e., those with high turnover intentions and poor job satisfaction in Sweden. Two research questions were addressed: (1) What characterize nurses who are discontent with their current job? and (2) How are nurses' discontent with their job associated with background and organizational workplace factors?

The knowledge contribution relates to the combination of variables and the associations between them. Furthermore, this study contributes knowledge about nurses' psychosocial working conditions in primary healthcare. These issues are important, considering that primary healthcare in Sweden and internationally is facing nursing shortages. Knowledge of these issues will be valuable for developing nursing retention and recruitment strategies.

## 2. Materials and Methods

### 2.1. Study Setting

This is a cross-sectional questionnaire study of registered nurses working in primary healthcare in Sweden. The Swedish primary healthcare system is divided into 21 different regions (previously known as “county councils”) and provides universal coverage for all citizens. Primary healthcare, often referred to as the “first line” of healthcare, manages a broad range of conditions that do not require specialist healthcare in hospitals or other specialized units, commonly including access to different professionals such as physicians, nurses, occupational therapists, and physiotherapists. In Sweden, primary healthcare consists of both public and private professionals; private professionals can include those with a contract with the region (which provides healthcare at the same costs as public healthcare) or those without regional contracts (which patients pay for themselves) [50].

Primary healthcare in Sweden employs about 25,000 people, and registered nurses make up 43% of the workforce [51]. Nurses are predominantly female. The role of the registered nurse in primary healthcare typically involves tasks such as handling medication, performing different tests on patients, documenting patient records, telephone counseling, and assessing and prioritizing a patient's need for healthcare. They also have a leading role in structuring nursing work in collaboration with other team members such as assistant nurses.

### 2.2. Data Collection

An invitation to participate in this study, along with an information letter and a questionnaire, were sent in March 2022 by Statistics Sweden (SCB) to a representative selection of registered nurses working in Sweden. This selection was made in consideration of geographic spread, and the population was identified through the 2020-2021 occupational registry and the national educational registry. The total population consisted of 109,475 registered nurses, and SCB randomly selected a sample of 8000 registered nurses who were invited to answer the survey. The information letter addressed ethical aspects, such as assuring confidentiality and that the respondents accepted participation in the study by answering the questionnaire. The letter also provided information about the study's aims, and that data collection included both the questionnaire and registry data. Respondents were informed that they could answer the questionnaire either online or by filling out the paper form sent with the invitational letter.

Three reminders were sent by post, and data collection closed in June 2022. In total, 2903 nurses answered the questionnaire, giving a 37.2% response rate. Of the respondents, 75.2% chose to answer the questionnaire online. Most of the nurses responded to the first invitation (33.4% of the total population).

A nonresponse analysis was performed by SCB, revealing that 48 of the nurses lacked a valid postal address or had protected identities, 21 declined participation with no reason given, 1 was prevented from participating (because of either language difficulties or disability), and 3 were labeled as “others” (because the returned questionnaire was unusable for some reason). The remainder of the nurses did not respond, and no reasons were provided.

In this study, we were particularly interested in the working conditions for nurses in primary healthcare; therefore, nurses who stated that they worked in a primary healthcare setting (*n* = 466) were selected. Inclusion criteria included working in Sweden, being a registered nurse, and stating currently working in primary healthcare.

### 2.3. The Questionnaire

The questionnaire was developed together with researchers at the Karolinska Institute in Sweden. This questionnaire has been used previously by Hagqvist et al. [52] and the 2022 version included 78 variables focusing on work environment issues. The questionnaire was developed for physicians, registered nurses, and assistant nurses working in various healthcare settings in Sweden. For this specific study, we restricted the analyses to nurses working in primary healthcare settings and chose to include a limited number of variables relating to sociodemographic characteristics, background factors, health-related variables (sickness presenteeism and perceived health), psychosocial working conditions (change fatigue, leadership climate, and social support from colleagues), and work-related outcomes (turnover intentions and job satisfaction).

The questionnaire data were linked to registry data collected from the national population registry for demographic information (age and sex).

### 2.4. Measures

This study investigated discontent with one's job. To this end, we created a new variable indicating discontent by combining respondents' ratings on turnover intentions and job satisfaction. Nurses who both had high rates of turnover intentions and low rates on job satisfaction were included.

We chose to include whether the nurses had a specialist degree, whether they had changed their job in the last 12 months from a region to a staffing agency, their main employer, work experience, whether they were working full or part time, their estimated working hours per week, whether they had a partner, and whether they had children living at home.

Age was categorized into four fairly evenly sized groups, and sex was categorized as male or female. For educational level, we chose to categorize the nurses as either registered nurses or specialist nurses (in which we also included midwives and radiology nurses).

The response options for their main employer were as follows: municipality, region, private employer with public funding, private employer with private funding, public employer, staffing agency, self-employed, or none of these, which included an open text response. These responses were categorized into five categories for this study: municipality, region, private employer with public funding, private employer with private funding, and others (which included the responses with the lowest number of respondents, i.e., those publicly employed, in staffing agencies, those self-employed, and those who answered that none of the response options applied to them). Swedish primary healthcare consists of public actors and two types of private actors: those having a contract with the region which provides healthcare at the same cost for citizens as public healthcare, and those without regional contracts, where the patients pay for the visits themselves [50]. In Sweden, the regions are the largest employers of healthcare staff, and this was the reference group in our analyses of main employer.

The options for estimated working hours per week were <30 h, 30–35 h, 36–40 h, 41–45 h, 46–50 h, 51–60, or >60 h per week. For this study, we chose to dichotomize at 40 h, dividing the respondent's answers into two groups: ≤40 h per week or >40 h per week (as 40 h of work per week is the standard for working full-time in Sweden).

#### 2.4.1. Turnover Intentions

Turnover intentions refer to an employee's willingness or intention to voluntarily quit their job [11]. The turnover intentions among the respondents were measured with one question: How often during the past 12 months have you considered applying for a new job? Five response options were provided on a Likert scale, ranging from *every day* to *never*.

#### 2.4.2. Job Satisfaction

Job satisfaction is defined as the extent to which a person reports overall satisfaction with their work [53]. For this study, job satisfaction was measured with one question: How satisfied or dissatisfied are you with your job? The five response options ranged from very satisfied to very dissatisfied.

#### 2.4.3. Discontent With Current Job

Based on the responses pertaining to turnover intentions and job satisfaction, we created a combined measure in order to investigate the characteristics of the respondents with high turnover intentions and low job satisfaction. The rationale for combining the two variables was to identify nurses who expressed high turnover intentions and low job satisfaction simultaneously, which potentially suggests a stronger predictor of being discontent with their job.

Specifically, we created a category consisting of those who rated their job satisfaction as very dissatisfied, fairly dissatisfied, or neither satisfied nor dissatisfied and rated their turnover intentions as having considered leaving their current work every day, a couple of times a week, or a couple of times a month. This newly created combination variable is referred to as “discontent with current job.” From this variable, we aimed to gain an improved understanding of the factors contributing to nurses' turnover and identify potential strategies to address these issues.

#### 2.4.4. General Health

In the questionnaire, the respondents were also asked how they perceived their health in general, rating on a five-point Likert scale ranging from *excellent* to *poor*.

#### 2.4.5. Sickness Presenteeism

Sickness presenteeism is described as “the first cousin of absenteeism that occurs when employees show up for work but because of mental or medical illness do not function productively or perform at 100%” ([54] p. 1).

One question in the questionnaire was used to investigate potential sickness presenteeism: “During the past 6 months, how many days in total have you gone to work when you should have called in sick?” We used three categories: those who stated they had no such days, those reporting 1–5 days of sickness presence, and those with more than 5 days for the past 6 months.

#### 2.4.6. Change Fatigue

Change fatigue is the perception that there are too many organizational changes taking place simultaneously, which may lead to exhaustion and the incapacity to support and adapt to further changes, regardless of whether they may be favorable [30]. The change fatigue scale by Bernerth et al. [27] is a validated measure originally developed to explore the impact of multiple organizational changes on employee's well-being, organizational commitment, and turnover intentions [27]. The scale contains items such as “the number of changes that are carried out in my workplace is overwhelming,” “I am sick of all the changes at work,” and “I would prefer a period of stability before another change is carried out in the organization.”

For this study, the measure was translated into Swedish and subsequently reviewed by bilingual colleagues and validated through a cognitive pretest inspired by cognitive interviewing [55]. This was performed by HF and JS, who tested the translated measure linguistically and semantically on four physicians and two nurses, respectively, who were asked to answer the translated measure while expressing their thoughts and interpretations of the questions out loud. After this, JS and HF discussed the findings together with PN and IS, which resulted in minor revisions and clarifications of a few terms in some of the questions.

The original change fatigue scale uses six items that are measured using a seven-point Likert-like scale ranging from 1 (“*strongly disagree*”) to 7 (“*strongly agree*”) [27]. In this study, the same six items were measured using a five-point Likert scale upon the advice of SCB after scrutinization of the questionnaire and the responses are as follows: (1) “strongly disagree,” (2) “disagree,” (3) “neutral,” (4) “agree,” and (5) “strongly agree.” Change fatigue scale was calculated as the sum of different items. Cronbach's alpha for change fatigue was 0.96, indicating the internal consistency of the scale.

#### 2.4.7. Leadership Climate

Leadership climate is described as how managers set an emotional tone and create mutual trust within the work environment [43]. Rather than rating one's managers' individual skills as a boss, leadership climate captures one aspect of the psychosocial work environment, thus referring to an organizational level rather than an individual level. This climate was measured with a ten-item instrument [56, 57], including statements such as: “I am clear about what my boss expects of me,” “I have sufficient authorization in relation to the responsibility I have,” and “my boss encourages me to participate in the arrangement of my work,” with five response options ranging from “yes, often” to “no, never” and including the option “not relevant.” The leadership climate scale was calculated as the sum of the items of the scale. Cronbach's alpha for leadership climate was 0.88.

#### 2.4.8. Social Support From Colleagues

The respondents rated perceived social support from colleagues with two questions from the Copenhagen Psychosocial Questionnaire, third version (COPSOQIII) measure: If you need it, do you get help and support from your colleagues? A five-point Likert scale was used, ranging from “*always*” to “*never*.” Cronbach's alpha for social support from colleagues was 0.77.

### 2.5. Data Analysis

When variables in a psychosocial scale (leadership climate, social support from colleagues, and change fatigue) were missing, a total score was calculated for that person if at least half of the questions of the scale were answered, and the missing variables were given the average score of the other variables in the scale [58]. The reliability of the psychosocial scales (leadership climate, social support from colleagues, and change fatigue) was estimated using Cronbach's alpha internal consistency coefficient.

The distribution of sample characteristics and discontent was estimated for all respondents. Descriptive statistics are presented as frequencies for categorical variables or mean values and standard deviations (SDs) for continuous variables. General health was dichotomized prior to the regression analysis (1 = *good health*, being either “excellent health,” “very good health,” or “good health” vs. 2 = *poor health*, being either “fairly good health” or “bad health”). The psychosocial scales (leadership climate, social support from colleagues, and change fatigue) were standardized with mean zero and SD one and were used as continuous variables prior to the regression analysis.

Logistic regression was used to identify the characteristics of those who had a high level of discontent with their current job. The logistic regression analysis was unadjusted in Model I and was adjusted for all confounders in Model II (sex, age, main employer, working time, general health, change fatigue, leadership climate, and social support from colleagues). Odds ratios (ORs) for high discontent were estimated with 95% confidence intervals. The ORs for discontent with one's current job are expressed per one SD increase for the psychosocial scales (mean, 0; SD, 1).

Results were considered statistically significant at *p* < 0.05 using two-tailed tests. Statistical analyses were performed using SPSS version 28.

### 2.6. Ethical Considerations

Study procedures and data collection were performed in accordance with the Helsinki Declaration [59]. Respondents were informed in the letter that participation was voluntary and confidential and that they had the right to withdraw their participation at any time. This study was approved by the Swedish Ethical Review Authority (Dnr 2022-03275-01; 2022-00310-02).

## 3. Results

### 3.1. Sample Characteristics

The characteristics of the respondents are presented in Table 1. This study included 466 respondents, mostly female nurses (*n* = 447; 95.9%). About half of the respondents were 50 years or older (*n* = 235; 50.4%). Of those who answered the questionnaire, 71.4% had an additional specialist education. Most respondents were employed in regions (*n* = 317; 68.3%), and 26% were employed in the private sector.

Although 51.6% of all respondents worked part time, 30.8% stated that they worked more than 40 h per week. In terms of general health, the nurses reported adequate health overall; 77.1% reported good, very good, or excellent health. However, regarding sickness presenteeism, 33.7% of the nurses reported being sick at work to some extent over the past 12 months.

The mean score for leadership climate was 30.3 on a scale ranging from 10 to 40, indicating that most of the nurses were fairly content in this regard. The perception of social support from colleagues was rated at a mean of 9.1 on a scale ranging from 2 to 10, which implies a high level of social support from colleagues. The mean score for change fatigue was 17.3 on a scale ranging from 6 to 30. This indicates that change fatigue is a feature experienced by nurses in primary healthcare.

### 3.2. Discontent With Current Job, Job Satisfaction, and Turnover Intentions

The results for discontent with one's current job, job satisfaction, and turnover intentions are presented in Table 2. For the primary outcome variable, discontent, 21.1% of the respondents were dissatisfied with their current job and had considered looking for a new job. The characteristics of these nurses and the associations with other variables were explored further through a regression analysis, as shown in Table 3.

### 3.3. Characteristics of Primary Care Nurses Who Are Discontent With Their Current Job

Crude and multivariable logistic regression models are presented in Table 3. In Model II, ORs are adjusted for all other variables in the table (sex, age, main employer, working time, general health, change fatigue, leadership climate, and social support from colleagues). Significant associations between discontent with one's current job and the leadership climate were demonstrated, with a positive leadership climate reducing nurses' discontent (OR = 0.39; 95% CI 0.28–0.55). Similarly, higher ratings for social support from colleagues were significantly associated with a lower score for discontent (OR = 0.52; 95% CI 0.38–0.72) and change fatigue was associated with higher discontent (OR = 2.18; 95% CI = 1.54–3.10). Those who reported working more than 40 h per week were more than twice as likely to be dissatisfied with their current job compared to those who reported working less than 40 h per week (OR = 2.13; 95% CI 1.14–3.98). Furthermore, respondents who reported poor health were almost four times as likely to experience discontent compared to those who reported good health (OR = 3.90; 95% CI 2.03–7.50).

## 4. Discussion

This study aimed to investigate working conditions (change fatigue, leadership climate, and social support from colleagues) and characteristics of primary care nurses who are discontent with their current job, i.e., they have both high turnover intentions and low job satisfaction. One in four nurses in primary care in Sweden is discontent with their current job. In terms of turnover intentions, 69.8% of nurses reported that they, to some extent over the past 12 months, had considered getting a new job and 13.8% rated their job satisfaction as being “fairly dissatisfied” or “very dissatisfied.” Nurses who were discontent with their job to a larger extent reported a poor leadership climate, less social support, more change fatigue, and poorer general health. Our finding suggests the importance of improving working conditions for nurses in primary healthcare in Sweden to increase their job satisfaction and willingness to stay at their current job. While turnover intentions are a proxy measure used to capture the feelings that lead to leaving a job, research shows that turnover intentions are related to an increased probability of actual resignation [11, 60]. In fact, the proportion of permanent staff on the team predicts the likelihood of leave rates [60]. Thus, creating attractive workplaces for nurses is essential to prevent continuous employee turnover that may strain both those who leave and those who remain. Limitations in the working environment for nurses have been increasingly highlighted, with reports of high workloads and excessive administrative work unrelated to patient care [61, 62].

We found that a positive leadership climate was associated with reduced discontent among nurses with their current job. Similar associations have been found in other studies of nurses in primary healthcare [63, 64]. These findings can be related to previous research on managerial leadership and its influence on employees' health and well-being (e.g., [65, 66]). For example, studies show that a supportive and relationship-oriented leadership (e.g., managers who express concern, support, and empathy for their staff) is important for employees' well-being at work, including job satisfaction and work engagement [67]. Nursing management can have a significant impact on the work climate, including improving job satisfaction, employee engagement, retention rates and overall staff well-being. Studies show that transformational leadership in nursing management is linked to higher job satisfaction and lower burnout rates among nurses [68]. Nursing managers who provide support, recognition, and feedback to their staff can boost morale and job satisfaction, thus creating a positive work climate and improving nurse well-being and job performance [69].

Experiencing social support from colleagues was another aspect that our study highlighted as an important factor for lower scores for discontent with one's current job. This finding is also in line with previous research [70–73]. It has been shown that supportive colleagues who respect and trust each other, facilitate work motivation, thus encouraging nurses to remain at work [72, 73]. Other positive work environmental aspects, such as recognition for the work that the nurses perform and autonomy and flexibility to find creative solutions for patients, can contribute to increased job satisfaction [70] and help prevent burnout [74].

Our study demonstrated that change fatigue and poor health were significantly associated with nurse dissatisfaction with their current job. Research demonstrates that change fatigue is associated with decreased job satisfaction [30, 75], as well as increased turnover and absenteeism among nurses [75]. Thus, perceptions that there have been too many organizational changes are one aspect that may negatively influence the possibility of sustainable nurse retention.

There was a trend in our study toward nurses working in regions being more dissatisfied with their current job compared with those working in municipalities or private practices with public funding. A Swedish report highlighted the difficulty in recruiting experienced professionals and professionals willing to work in the regions [2]. Notably, we found that almost one-third of the nurses in our study reported working more than 40 h per week (the Swedish full-time norm), which was another aspect related to dissatisfaction. More than half of the nurses were supposed to work part-time according to their responses as to whether they worked full-time or part-time. Other studies have demonstrated that overtime work is associated with poor nursing performance and decreased patient safety [61], low job satisfaction [60], and an increased risk that the nurse will be afflicted with occupational hazards and injuries [76].

The findings from this study have policy implications for how healthcare organizations and nursing management structure the work of nurses in primary healthcare, how they can attract staff to these workplaces, and how to facilitate nursing retention. High turnover rates may be harmful to healthcare quality if skilled nurses often leave, and nursing staff includes a high proportion of novices. Nurses' working environment affects the quality of care that they provide to their patients [77]; thus, the quality of their work environment may also affect patient safety. Ensuring that nurses are willing to remain at their workplace and their profession is essential for patient safety and to meet healthcare needs [78]. This is a pressing issue and a challenging task for healthcare organizations and decision-makers. What we do not know, and thus could be of interest to explore further, is what “solutions” for the sustainable retention of nurses are used in practice and considered beneficial for improving their psychosocial working conditions. Specifically, future research is needed on organizational key achievements rather than interventions at the individual level.

There are several methodological issues to consider when interpreting the findings. We used a quantitative research method for this study. The aim was to investigate a population of nurses at one particular point in time, which made a cross-sectional survey appropriate. This type of research makes it possible to reach many individuals in a short time in an inexpensive manner. However, it does not allow for causal inference, as cross-sectional studies can only point to statistical associations between variables [79]. Another limitation was the relatively low response rate and the inability to analyze nurses' reasons for not participating. This means that the generalizability of the findings is somewhat restricted. SCB distributed the questionnaires and provided the researchers with a technical report, including a dropout analysis for the full sample, including nurses in all types of settings—not solely primary healthcare. The response rate for the questionnaire among these nurses was 37.3% (2903 participating respondents). A comparison to the target population showed that there were relatively more nurses born in Nordic countries or with a Swedish background among the survey respondents and that respondents tended to be a little older than the target population and were less likely to be newly graduated nurses.

We lack information about how many primary healthcare nurses were included in the total population. Therefore, we do not know the response rate for this group. However, there are no indications that nurses in primary healthcare would be either more or less likely to answer a questionnaire on psychosocial working conditions than nurses in other settings. As in most research, there is a risk that those who participate are the ones most interested in the topic or most capable of responding. Research has established that more motivated and opinionated people are more likely to respond to surveys [80] and that a lack of motivation, high workload, bad timing, and inaccurate addresses may cause dropouts and nonresponses [81]. Hence, it is plausible that the respondents in this study were either more positive or more negative to the issues we investigated.

Furthermore, the sex distribution in this study is overrepresented by female respondents. However, this is not surprising because the healthcare sector, and nursing in particular, is heavily dominated by females [82]. In 2022, 87% of the nursing population in Sweden were women [82]. Other sampling strategies and analytical methods are required to obtain a clearer gender perspective and to compare the perceptions of male and female nurses. This was not the purpose of the current study but might be of interest in future studies.

In terms of generalizability, we can conclude that the findings of this study are in line with previous research within this area. However, the Swedish healthcare system is part of a comprehensive welfare system with universal coverage for all citizens, i.e., it provides care to anyone in need of it. This might lead to other types of demands on nurses as citizens in Sweden might be more prone to seeking care. Thus, our findings may to some extent be less generalizable to countries with other types of welfare systems, such as the American liberal system in which the state emphasizes the individuals' responsibilities and the need for private health insurance is greater. Although this was not a comparative study between systems or countries, nurses' working conditions may differ depending on which system they work in, and the generalizability of our study's findings, therefore, is mainly related to similar comprehensive healthcare systems.

## 5. Conclusions

In conclusion, this study found that nurses' discontent with their current job (i.e., high turnover intentions combined with low job satisfaction) is associated with the leadership climate, social support from colleagues, working more than 40 h per week, change fatigue, and poor health. Specifically, experiencing poor health, change fatigue, or working more than 40 h per week were associated with being more discontent, while the perception of a good leadership climate and supportive colleagues were associated with decreased rates of discontent. The retention and recruitment of nurses in primary healthcare are essential to ensure stability in the movement of personnel and to prevent the need for nurses to work more than 40 h per week, which in this study turned out to be associated with discontent.

This knowledge may facilitate the identification of potential problems in need of solutions that could benefit nurses in terms of improved working conditions, patients in terms of potentially increased quality of care, and organizations who are interested in strategies for retaining their staff at work.

## Figures and Tables

**Table 1:** Characteristics of the survey respondents among nurses in primary healthcare.

**Variable**	**Number**	**Mean ± SD**	**Frequency (%)**
Sex	466		
Male			19 (4.1)
Female			447 (95.9)
Age	466		
≤40 years			111 (23.8)
41–50 years			120 (25.8)
51–60 years			130 (27.9)
≥61 years			105 (22.5)
Educational level	464		
Registered nurse			137 (28.6)
Specialist nurse			327 (71.4)
Have changed job in the last 12 months from region to staffing agency	464		
Yes			29 (6.3)
No			435 (93.8)
Main employer	464		
Region			317 (68.3)
Municipality			12 (2.6)
Private sector (publicly funded)			92 (19.8)
Private sector (privately funded)			27 (5.8)
Others			16 (3.4)
Working experience as a nurse	466		
<5 years			40 (8.6)
5–10 years			45 (9.7)
10–15 years			87 (18.7)
>15			294 (63.1)
Working full time or part time	465		
Full time			225 (48.4)
Part time			240 (51.6)
Work time/week	465		
≤40 h			322 (69.2)
>40 h			143 (30.8)
Having a partner	466		
Yes			414 (88.8)
No			52 (11.2)
Having children living at home	461		
Yes			253 (54.9)
No			208 (45.1)
General health	463		
Excellent			38 (8.2)
Very good			142 (30.7)
Good			177 (38.2)
Fairly good			78 (16.8)
Poor			28 (6.0)
Sickness presence (in the last 6 months)	448		
None			297 (66.3)
1–5 days			92 (20.5)
>5 days			59 (13.2)
Change fatigue	465	17.3 ± 6.6	
Leadership climate	461	30.3 ± 6.2	
Social support from colleagues	458	9.1 ± 1.3	

*Note:* When data are missing, the numbers do not sum up to 466 due to internal dropout.

**Table 2:** Descriptive statistics of job satisfaction, turnover intention, and the primary outcome—discontent with current job, among primary healthcare nurses.

**Study variables**	**Number**	**Frequency (%)**
Job satisfaction	463	
(1) Very satisfied		113 (24.4)
(2) Fairly satisfied		236 (51.0)
(3) Neither satisfied nor dissatisfied		50 (10.8)
(4) Fairly dissatisfied		53 (11.4)
(5) Very dissatisfied		11 (2.4)
Turnover intentions (how often during the last 12 months have you considered getting a new job)	463	
(1) Every day		40 (8.6)
(2) Once/a couple of times a week		63 (13.6)
(3) Once/a couple of times a month		73 (15.8)
(4) Once/a couple of times in the last 12 months		147 (31.7)
(5) Never		140 (30.2)
Discontent with the current job	460	
No		363 (78.9)
Yes		97 (21.1)

*Note:* When data are missing, the numbers do not sum up to 466. Only 460 participants responded simultaneously to the two variables.

**Table 3:** Logistic regression of discontent with the current job among nurses in primary healthcare.

**Variables**	**Model I (crude)**			**Model II (multivariable)**		
	**OR**	**95% CI**	***p*** ** value**	**OR**	**95% CI**	***p*** ** value**
Sex						
Male	1			1		
Female	1.45	0.41–5.06	0.565	1.120	0.23–5.42	0.888
Age						
≤40 years	1			1		
41–50 years	0.60	0.32–1.11	0.100	0.45	0.20–1.05	0.066
51–60 years	0.64	0.35–1.16	0.137	0.34	0.15–0.78	0.011
≥61 years	0.50	0.26–0.98	0.043	0.25	0.10–0.66	0.005
Main employer						
Region	1			1		
Municipality	0.26	0.03–2.07	0.205	0.25	0.03–2.25	0.218
Private (publicly funded)	0.24	0.11–0.54	<0.001	0.25	0.09–0.75	0.013
Private (privately funded)	0.66	0.24–1.80	0.416	1.05	0.26–4.16	0.948
Others	0.67	0.19–2.41	0.539	0.84	0.10–7.22	0.877
Work time/week						
<40 h	1			1		
>40 h	2.67	1.68–4.24	<0.001	2.13	1.14–3.98	0.018
General health						
Good health (excellent, very good, or good)	1			1		
Poor health (fairly good or bad health)	4.42	2.71–7.19	<0.001	3.90	2.03–7.50	<0.001
Change fatigue	2.48	1.89–3.24	<0.001	2.18	1.54–3.10	<0.001
Leadership climate	0.32	0.24–0.42	<0.001	0.39	0.28–0.55	<0.001
Social support from colleagues	0.44	0.35–0.56	<0.001	0.52	0.38–0.72	<0.001

## Data Availability

The questionnaire data used in this study are restricted by the Swedish Ethical Review Authority in order to protect patient privacy. Furthermore, the respondents have consented to participate in studies conducted by this research group, and we thus lack their consent to share the data.
